# [^18^F]FMISO PET/CT as a preoperative prognostic factor in patients with pancreatic cancer

**DOI:** 10.1186/s13550-019-0507-8

**Published:** 2019-05-09

**Authors:** Tomohiko Yamane, Masayasu Aikawa, Masanori Yasuda, Kenji Fukushima, Akira Seto, Koujun Okamoto, Isamu Koyama, Ichiei Kuji

**Affiliations:** 1 Department of Nuclear Medicine, Saitama Medical University International Medical Center, 1397-1 Yamane, Hidaka, 350-1108 Japan; 2 Department of Gastroenterological Surgery, Saitama Medical University International Medical Center, 1397-1 Yamane, Hidaka, 350-1108 Japan; 3 Department of Diagnostic Pathology, Saitama Medical University International Medical Center, 1397-1 Yamane, Hidaka, 350-1108 Japan

**Keywords:** Hypoxia, Pancreas, F-18 fluoromisonidazole (F-18 FMISO), HIF-1 alfa, Surgery, Operation, Pancreatectomy

## Abstract

**Background:**

While [^18^F]fluoromisonidazole (FMISO), a representative PET tracer to detect hypoxia, is reported to be able to prospect the prognosis after treatment for various types of cancers, the relation is unclear for pancreatic cancer. The aim of this study is to assess the feasibility of [^18^F]FMISO PET/CT as a preoperative prognostic factor in patients with pancreatic cancer.

**Methods:**

Patients with pancreatic cancer who had been initially planned for surgery received [^18^F]FMISO PET/CT. Peak standardized uptake value (SUV) of the pancreatic tumor was divided by SUVpeak of the aorta, and tumor blood ratio using SUVpeak (TBRpeak) was calculated. After preoperative examination, surgeons finally decided the operability of the patients. TBRpeak was compared with hypoxia-inducible factor (HIF)-1α immunohistochemistry when the tissues were available. Furthermore, correlation of TBRpeak with the recurrence-free survival and the overall survival were evaluated by Kaplan-Meyer methods.

**Results:**

We analyzed 25 patients with pancreatic adenocarcinoma (11 women and 14 men, median age, 73 years; range, 58–81 years), and observed for 39–1101 days (median, 369 days). Nine cases (36.0%) were identified as visually positive of pancreatic cancer on [^18^F]FMISO PET/CT images. TBRpeak of the negative cases was significantly lower than that of the positive cases (median 1.08, interquartile range (IQR) 1.02–1.15 vs median 1.50, IQR 1.25–1.73, *p* < 0.001), and the cutoff TBRpeak was calculated as 1.24. Five patients were finally considered inoperable. There was no significant difference in TBRpeak of inoperable and operable patients (median 1.48, IQR 1.06–1.98 vs median 1.12, IQR 1.05–1.21, *p* = 0.10). There was no significant difference between TBRpeak and HIF-1α expression (*p* = 0.22). The patients were dichotomized by the TBRpeak cutoff, and the higher group showed significantly shorter recurrence-free survival than the other (median 218 vs 441 days, *p* = 0.002). As for overall survival of 20 cases of operated patients, the higher TBRpeak group showed significantly shorter overall survival than the other (median survival, 415 vs > 1000 days, *p* = 0.04).

**Conclusions:**

[^18^F]FMISO PET/CT has the possibility to be a preoperative prognostic factor in patients with pancreatic cancer.

**Electronic supplementary material:**

The online version of this article (10.1186/s13550-019-0507-8) contains supplementary material, which is available to authorized users.

## Background

For the viewpoint of image-based diagnosis, operability for pancreatic cancer has been evaluated by local tumor size or involvement of surrounding organs detected by computed tomography (CT) or magnetic resonance imaging (MRI) [1]. Prognosis of pancreatic cancer is, however, generally poor and we often experience many cases of recurrence after surgery. Therefore, constructing strict decision-making is quite important. Recently, positron emission tomography (PET) used 2-deoxy-2-[^18^F]fluoro-D-glucose ([^18^F]FDG) is widely utilized for initial staging, re-staging, and predicting the prognosis for malignant tumor as well as for pancreatic cancer [2, 3]. However, pancreatic cancer is well known to be associated with diabetes mellitus or pancreatitis [4, 5]. [^18^F]FDG is a marker of glucose metabolism and occasionally affected for the patients with diabetes mellitus as false negative [6] or patients with pancreatic inflammation as false positive [7].

Pancreatic cancer is known that it includes hypoxic fraction in the tumor. Hypoxic stress activates a variety of reactions like hypoxia-inducible factor (HIF) pathway, and they cause tumor invasion or metastasis to another organ or lymph node [8, 9]. Therefore, investigating tumor hypoxia is very important to evaluate the prognosis. While several ways to evaluate hypoxia is known such as oxygen electrode, MRI [10] or near-infrared diffuse optical imaging [11], PET with hypoxic tracer is a noninvasive method with high repeatability [12, 13]. Among several types of PET tracers that can visualize hypoxia, [^18^F]fluoromisonidazole ([^18^F]FMISO) is one of the representative tracers. [^18^F]FMISO can visualize tumor hypoxia in many kinds of cancers, and recently it is known to be related to prognosis after the treatment such as in head and neck cancer [14], breast cancer [15], and glioblastoma [16]. However, there are few papers regarding hypoxic PET and pancreatic cancer [17–19], and nothing is referring to the prognosis.

The aim of this study is to test the feasibility of [^18^F]FMISO PET/CT as a preoperative prognostic factor in patients with pancreatic cancer.

## Materials and methods

### Patients

Our institutional review board approved the protocol of this study (Registration #14-110). From March 2015 to March 2018, we enrolled 26 patients with pancreatic cancer who had been initially planned for surgery. Inclusion criteria are the following: a pancreatic cancer patient who was referred to our surgery department, was 20-years old or older, and agreed to attend this study by written informed consent. We excluded a patient who was pregnant or who had the possibility of pregnancy and patient who was difficult to acquire PET/CT due to the physical condition such as unbearable pain. Patients were followed-up until November 2018 as much as possible. Recurrences after operations were evaluated by conventional methods including CT, MRI or [^18^F]FDG PET/CT.

### [^18^F]FMISO-PET/CT acquisition

Based on the previously described procedure [20], [^18^F]FMISO was synthesized in our hospital. Precisely 150 min after intravenous administration of 7.4 MBq/kg [^18^F]FMISO, PET/CT images were acquired by Biograph 16 (Siemens, Erlangen, Germany). The dose of syringes before and after [^18^F]FMISO administration was counted, and the correct injection dose was calculated. Images including entire pancreas were acquired for 20 min without any bed movement and reconstructed in a 168 × 168 × 32 matrix with 3.0 × 3.0 × 5.0 mm voxel size using ordered subset expectation maximization with iteration 3 subset 8 with Gaussian filter of 8-mm full width at half maximum. The attenuation correction was performed by transmission CT using the following parameters: slice thickness, 5 mm; tube rotation, 0.5 s; table speed, 10 mm/rotation; pitch, 1:1; 120 keV; and 50 mAs.

Two experienced nuclear medicine physicians (T.Y. and I.K.) evaluated [^18^F]FMISO uptake on tumor which can be visually identified and decided as positive or negative by discussion. The tumor that showed higher uptake than the surrounding normal pancreatic tissue was considered as positive. Three-dimensional spheroidal regions of interest (ROI) were drawn to cover the pancreatic tumor, and three-dimensional spherical ROI of a 10-mm diameter were drawn in the descending aorta on the same axial image to evaluate each standardized uptake value (SUV). SUVpeak of the tumor was divided by SUVpeak of the aorta, and we defined tumor blood ratio using SUVpeak (TBRpeak) as the quantitative value. SUVpeak was defined as the mean value of the voxels within a fixed sphere of 1-cm^3^ volume (approximately 22 voxels) centered on the hottest area of the ROI over the tumor and the aorta. Method for ROI placement was illustrated in Additional file 1: Figure S1. In case the tumor was difficult to identify on [^18^F]FMISO PET/CT alone, we set the ROI of the tumor by visually comparing side by side with the other modalities of enhanced CT, enhanced MRI, or [^18^F]FDG PET/CT. We used Syngo.via VA30A (Siemens, Erlangen, Germany) for the image analysis.

### Surgery and histopathological analysis

Based on the thorough discussion of patients’ information including enhanced CT and the other examinations except for [^18^F]FMISO PET/CT, surgeons finally decided their operability.

The expression of HIF-1α in the tumor was measured by immunohistochemistry for the patients who underwent tumor resection. For the patients who did not undergo surgery, tissue sections after biopsies were also used for HIF-1α immunohistochemistry when the tissues are available.

After deparaffinization, antigen retrieve was performed by boiling the section for 20 min at 98 °C in EnVision FLEX Target Retrieval Solution, High pH (Dako, Glostrup, Denmark) using PT Link (Dako). Subsequently, the tissues were incubated for 60 min with a monoclonal antibody for HIF-1α (ab210073, Abcam plc, Cambridge, United Kingdom) diluted to 1:100 in REAL Antibody Diluent (Dako). The endogenous enzyme was blocked with FLEX Peroxidase Block (Dako) for 10 min. For visualization, the sections were incubated for 30 min with FLEX/HRP (Dako) followed by a 10-min treatment by FLEX DAB+ (Dako). Furthermore, the sections were counterstained with Mayer’s hematoxylin for 3 min.

HIF-1α was evaluated by an experienced pathologist (M.Y.) without information of PET/CT and the other clinical information. The intensity of HIF-1α staining in the nuclei was semi-quantitatively categorized into no staining (score 0), weak staining (score 1), moderate staining (score 2), and strong staining (score 3). These scores were dichotomized into low HIF-1α expression (score 0 and 1) and high HIF-1α expression (score 2 and 3).

### Statistical analysis

Mann-Whitney-Wilcoxon test was used to compare 2 groups of TBRpeak on visual assessment, operability, and HIF-1α expressions. Kruskal-Wallis log-rank test was used to evaluate the difference of 4 categories of HIF-1α expressions. After dichotomization into positive or negative by the visual assessment of the nuclear medicine physicians, the results were compared with TBRpeak. The cutoff TBRpeak of visibility was determined at the maximum value of the sum of sensitivity and specificity on receiver operating characteristics (ROC) curve. An area under the curve (AUC) of ROC analysis was also calculated, and 0.9 or more of AUC was defined as high accuracy. The patients were dichotomized into high- or low-TBRpeak groups by the cutoff. For the patients who received operation, recurrence-free survivals (RFS) of the 2 groups were analyzed using Kaplan-Meier method, respectively. RFS was calculated by the days from [^18^F]FMISO PET/CT was performed to recurrence was diagnosed. Moreover, overall survivals (OS) were also analyzed for all patients and for patients who received operation only. OS were calculated by the days from [^18^F]FMISO PET/CT was performed to the patient died. For patients who are alive at the time of last follow-up, RFS and OS were censored of the day of the last follow-up. Median survivals and the 95% confidence interval (CI) were calculated, and these survival difference between high and low TBRpeak were compared by log-rank test.

A *p* value of less than 0.05 was considered to be significant in any statistical analysis. For the numbers, median and range, or median and interquartile range (IQR) were shown. For the statistical analysis, we used a statistical software Prism 8 (GraphPad Software, Inc., La Jolla, CA, USA).

## Results

### Patients

One patient out of 26 participants was finally diagnosed as solid-pseudopapillary neoplasm. Therefore, we excluded the patient and totally analyzed 25 patients with pancreatic adenocarcinoma (11 women and 14 men, median age, 73 years; range, 58–81 years). Observation period was 39–1101 days (median, 369 days). The tumor size ranged from 15.5 to 43.0 mm (median, 21.2 mm). Actual injected dose of [^18^F]FMISO ranged from 265 to 579 MBq (median, 417 MBq). Patient characteristics are shown in Additional file 2: Table S1 and flow diagram of the patients is shown in Additional file 3: Figure S2.

### Visual assessment

**Fig. 1:**
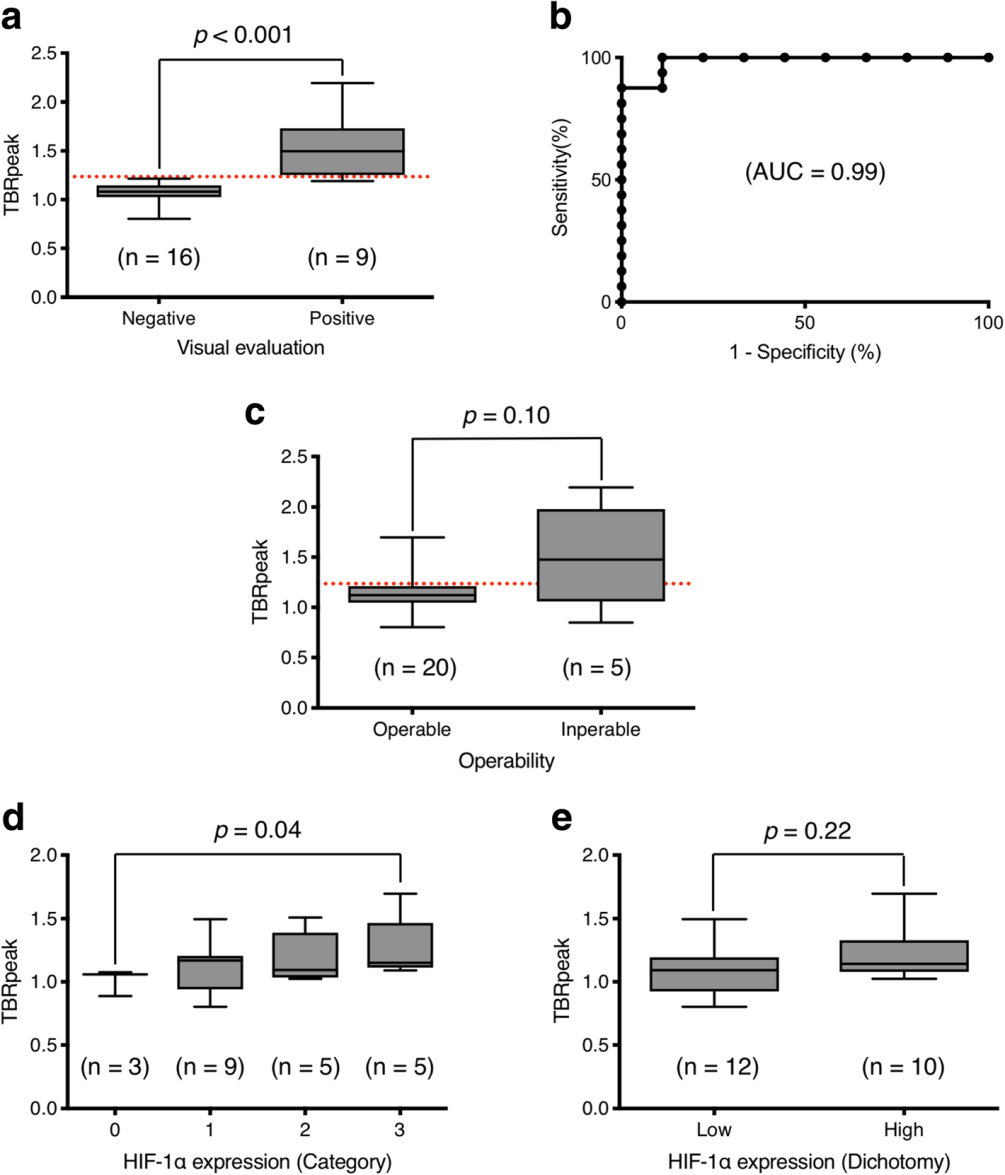
Box plots of TBRpeak by the results of visual evaluation of [^18^F]FMISO PET/CT (**a**), TBRpeak of operable and inoperable patients (**c**), TBRpeak by HIF-1α immunohistochemical expressions evaluated by 4 categories (0, 1, 2, and 3) (**d**), and 2 categories (low and high) (**e**). Red horizontal dot lines indicate TBRpeak = 1.24 calculated by the receiver operating characteristic analysis (**b**). Lines on whiskers and boxes in box blots indicate maximum, upper quartile, median, lower quartile, and minimum, respectively

After evaluation by the 2 nuclear medicine physicians, 9 cases (36.0%) were considered visually positive. Mean TBRpeak of the negative cases was significantly lower than that of the positive cases (median 1.08, IQR 1.02–1.15 vs median 1.50, IQR 1.25–1.73, *p* < 0.001), and the cutoff TBRpeak was calculated as 1.24 by the results of ROC analysis with the AUC of 0.99 (high accuracy) (Fig. 1a, b).

### Operability

As a result of thorough clinical examinations other than [^18^F]FMISO PET/CT, five patients were finally considered inoperable. There was no significant difference between inoperable and operable patients (median 1.48, IQR 1.06–1.98 vs median 1.12, IQR 1.05–1.21, *p* = 0.10) (Fig. 1c).

### HIF-1α expression

**Fig. 2:**
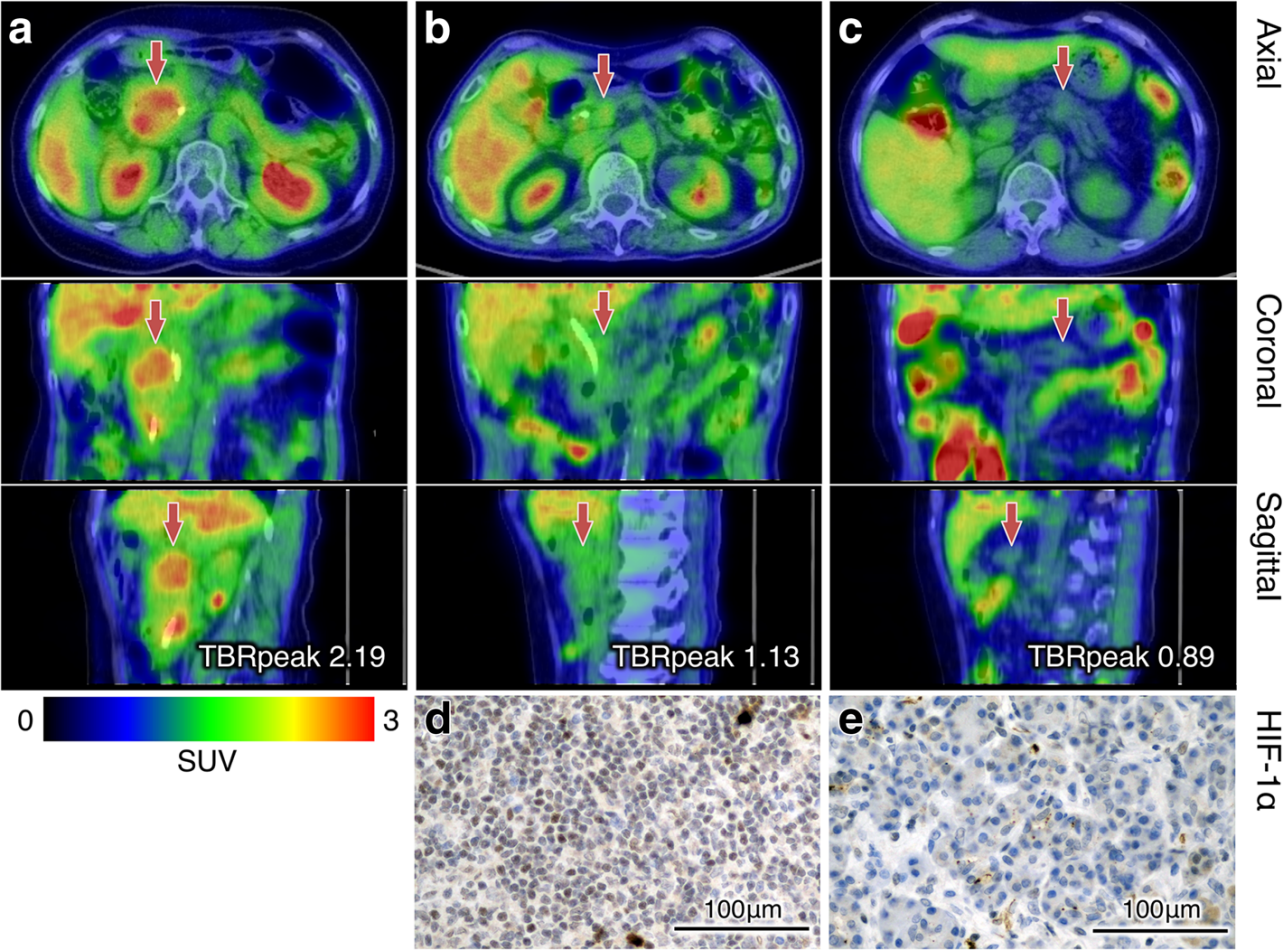
Representative [^18^F]FMISO PET/CT images (**a**, **b**, **c**) and HIF-1α immunohistochemical images (**d**, **e**) of pancreatic cancer. A color bar from 0 to 3 indicates standardize uptake value (SUV) of fused [^18^F]FMISO images. The first case with increased [^18^F]FMISO uptake on the primary tumor (**a**, arrows) was finally decided as inoperable. The second case with moderate [^18^F]FMISO uptake (**b**, arrows) received operation, and the immunohistochemistry shows strong HIF-1α expression (**d**). The third case with low [^18^F]FMISO uptake (**c**, arrows) also received operation, and the immunohistochemistry shows no apparent HIF-1α expression (**e**)

We evaluated HIF-1α expressions of 20 patients who underwent surgery plus 2 additional inoperable patients whose tissues of biopsy were available for immunohistochemistry. While there was a significant difference of TBRpeak between category 0 and 3 (*p* = 0.04), no significant difference was observed among all the groups by Kruskal-Wallis log-rank test (*p* = 0.32). Although the median of TBRpeak in the low-HIF-1α expression group (category 0 and 1) was lower than in the high-HIF-1α expression group (category 2 and 3), there was no significant difference (*p* = 0.22). Figure 1d, e shows the boxplots of TBRpeak by HIF-1α expression, and Fig. 2 shows the images of [^18^F]FMISO PET/CT and HIF-1α immunohistochemistry of representative cases.

### Survival curves

**Fig. 3:**
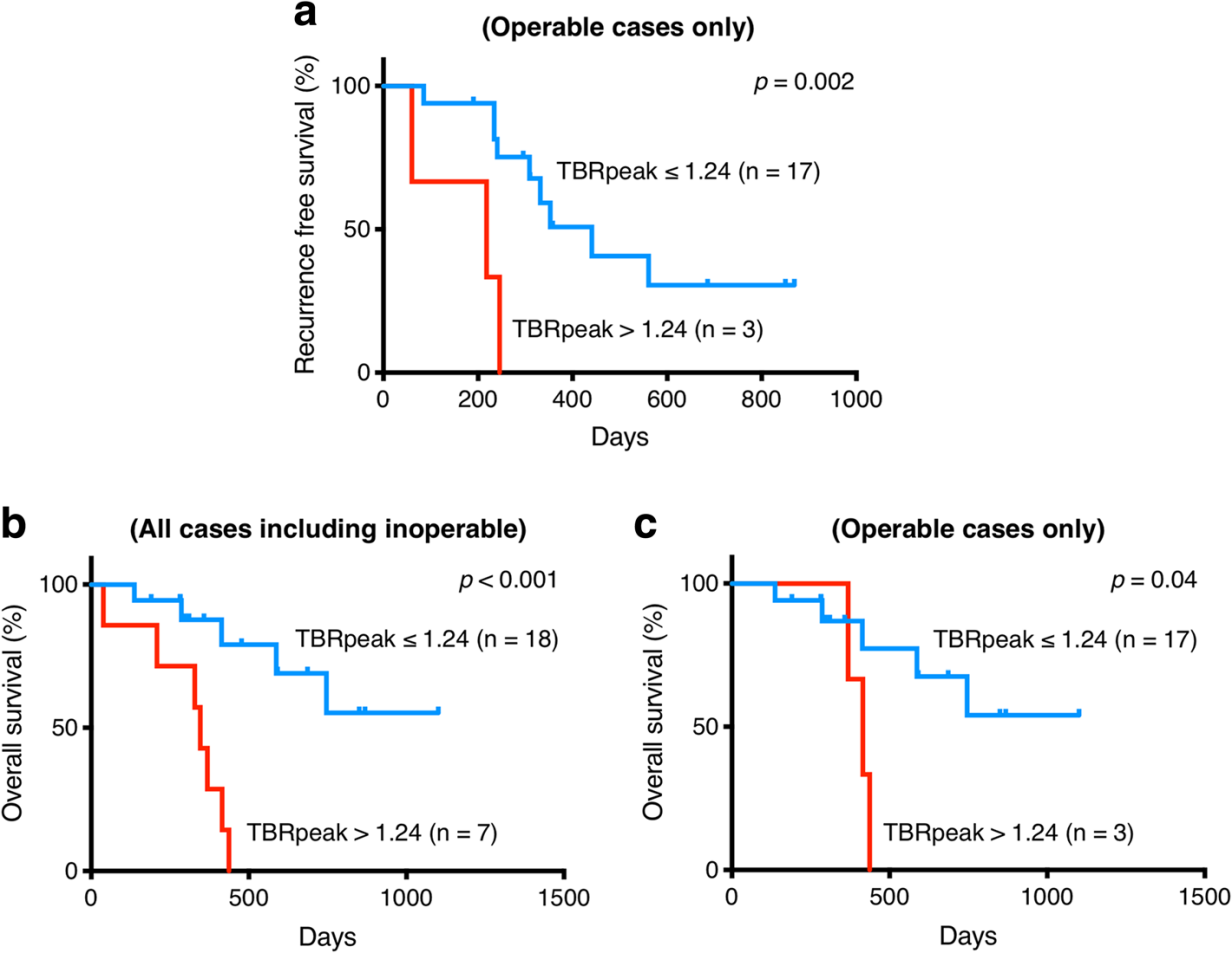
Kaplan-Meyer survival curves about the recurrence-free survival rate (**a**) and the overall survival rates of low-TBRpeak (blue lines) and high-TBRpeak (red lines). For the overall survival, curves of all cases including inoperable (**b**) and of only operable cases (**c**) are shown. TBR tumor blood ratio

The patients were dichotomized at the cutoff TBRpeak of 1.24, and the high-TBRpeak group showed significantly shorter RFS than the low-TBRpeak group (median survival, 218 (IQR 60–246) vs 441 (IQR 309–) days, *p* = 0.002) (Fig. 3a).

As for OS of all 25 cases, the high-TBRpeak group showed significantly shorter OS than low-TBRpeak group (median survival, 346 (IQR 208–415) vs > 1000 (IQR 587–) days, *p* < 0.001) (Fig. 3b). As for 20 cases who received operation, the high-TBRpeak group also showed shorter OS than low-TBRpeak group (Median survival, 415 (IQR 369–437) vs > 1000 (IQR 587–) days, *p* = 0.04) (Fig. 3c).

## Discussion

Pancreatic cancer was identified by [^18^F]FMISO PET/CT on 36.0% of the patients in this study, and the patients whose pancreatic cancer is identified tended to show the recurrences in their early stage and tended to have poor prognoses. Therefore, higher [^18^F]FMISO uptake especially visual identification of the tumor on [^18^F]FMISO PET/CT can be considered as an important prognostic factor.

Although inoperable cases generally showed higher TBRpeak, there was no statistically significant difference between operable and inoperable cases. Even small pancreatic cancer occasionally invades into surrounding tissue like the vascular system and results in inoperability. PET/CT generally underestimates these kinds of small lesions. Moreover, apparent inoperable cases have been eliminated for the entry to this study in advance. Therefore, no significant difference may have been observed.

HIF is a transcription factor which is activated when the intracellular circumstance turned to hypoxia, and it is known to be a strong prognostic factor in pancreatic cancer [21]. Some papers report that the overexpression of HIF-1α is significantly related to the increased [^18^F]FMISO uptake on the various kinds of malignant tumors [22, 23]. Although high TBRpeak of [^18^F]FMISO tended to show high expression of HIF-1α in the present study, there was no significant association between the [^18^F]FMISO uptake and the expression of HIF-1α. This might have been also caused by partial volume effect: the small tumor size in comparison with the other study treating tumors in the brain or the head and neck. In addition, we should note that there are some kinds of controversy over the relationship between hypoxia and HIF expressions [24, 25]. Therefore, this result does not deny the relationship between [^18^F]FMISO and hypoxia.

A case with higher [^18^F]FMISO uptake in the pancreatic cancer showed early recurrence after surgery. Therefore, [^18^F]FMISO PET/CT has the potential to be able to prospect the case that has the possibility to show early recurrence and need additional treatments after surgery. In addition, the results of [^18^F]FMISO PET/CT was also related to OS significantly. To our knowledge, this is the first paper that shows the relationship between [^18^F]FMISO PET/CT and the prognosis of pancreatic cancer. As new treatment methods and therapeutic agents are expected to improve the prognosis of pancreatic cancer [26–29], proper diagnostic methods are required to prospect the accurate prognosis for patients with pancreatic cancer. [^18^F]FMISO PET/CT, therefore, can be a novel prognostic biomarker for pancreatic cancer.

In the present study, we injected 7.4 MBq/kg of [^18^F]FMISO and the additional effective dose equivalent was estimated at approximately 5–10 MBq [30]. This dose injected was decided based on the previous [^18^F]FMISO PET/CT studies about pancreatic or lung cancers which are affected by respiratory motion [17]. In addition, shorter acquisition time was required because patients with pancreatic cancer are generally low-performance status. Because the patients with pancreatic cancer have already received dynamic enhanced CT and [^18^F]FDG-PET/CT for their initial staging, reduction of radiation exposure should be considered when we perform additional studies. The optimal dose should be considered in further studies.

There are some limitations to this study. At first, [^18^F]FMISO has the characteristic that shows low tumor contrast with the surrounding tissues. Moreover, pancreatic head cancer is occasionally difficult to discriminate with physiological uptake in the bile duct because [^18^F]FMISO is majorly excreted from the hepatobiliary system [30]. Although [^18^F]FMISO is a representative tracer for hypoxia imaging and has been used in many studies, novel tracers are expected to overcome the lipophilicity that would be the cause of low contrast and bile duct excretion. The other 2-nitroimidazoles tracer of [^18^F]fluoroazomycin arabinoside, known as [^18^F]FAZA, is reported to have more hydrophilic nature than [^18^F]FMISO, and it shows higher tumor background ratio [31]. Moreover, [^18^F]-2-(2-nitro-1[H]-imidazol-1-yl)-N(2,2,3,3,3-pentafluoropropyl)-acetamide, usually described as [^18^F]EF5, is noted to be excreted mainly via kidney [32]. These tracers have the possibility to evaluate hypoxia in pancreatic cancer more precisely. Further studies by these tracers are expected to prospect their prognosis in detail. In the present study, we set ROIs manually by nuclear medicine physicians even in the cases when [^18^F]FMISO uptake is invisible on the pancreatic tumor. Although we referred another modality to identify the pancreatic tumor, strict image-registrations were not performed. Therefore, the possibilities of misregistration also can be one of the limitations.

## Conclusion

Increased [^18^F]FMISO uptake in the preoperative pancreatic cancer is majorly related to the tumor hypoxia, and the tumor-blood ratio is correlated to the RFS and the OS. [^18^F]FMISO PET/CT has the possibility to be a preoperative prognostic factor in patients with pancreatic cancer.

## Additional files

Additional file 1: **Figure S1.** Three-dimensional region of interest (ROI) replacement. SUVpeak of the tumor (arrows) was divided by SUVpeak of the aorta (arrowheads), and we defined tumor-blood ratio using SUVpeak (TBRpeak) as the quantitative value. SUVpeak was defined as the mean value of the voxels within a fixed sphere of 1-cm^3^ volume centered on the hottest area of the ROI over the tumor. SUV, standardized uptake value. (PDF 221 kb)

Additional file 2: **Table S1.** Patients’ characteristics. (PDF 86 kb)

Additional file 3: **Figure S2.** Flow diagram of the patients. (PDF 155 kb)

## Abbreviations

[^18^F]FDG: 2-deoxy-2-[^18^F]fluoro-D-glucose
[^18^F]FMISO: [^18^F]fluoromisonidazole
AUC: Area under the curve
CI: Confidence interval
CT: Computed tomography
HIF: Hypoxia-inducible factor
IQR: Interquartile range
MRI: Magnetic resonance imaging
OS: Overall survival
PET: Positron emission tomography
RFS: Recurrence-free survival
ROC: Receiver operating characteristic
ROI: Regions of interest
SUV: Standardized uptake value
TBRpeak: Tumor blood ratio using SUVpeak (SUVpeak of the tumor divided by SUVpeak of the aorta)
