# Supplementary material for: [18F]FMISO PET/CT as a preoperative prognostic factor in patients with pancreatic cancer
Source: EJNMMI Res. 2019 May 9;9:39. doi: 10.1186/s13550-019-0507-8 (PMC6509312; doi:10.1186/s13550-019-0507-8)
Supplement: Supplementary file 1 — Figure S1. Three-dimensional region of interest (ROI) replacement. SUVpeak of the tumor (arrows) was divided by SUVpeak of the aorta (arrowheads), and we defined tumor-blood ratio using SUVpeak (TBRpeak) as the quantitative value. SUVpeak was defined as the mean value of the voxels within a fixed sphere of 1-cm3 volume centered on the hottest area of the ROI over the tumor. SUV, standardized uptake value. (PDF 221 kb) [file 13550_2019_507_MOESM1_ESM.pdf]

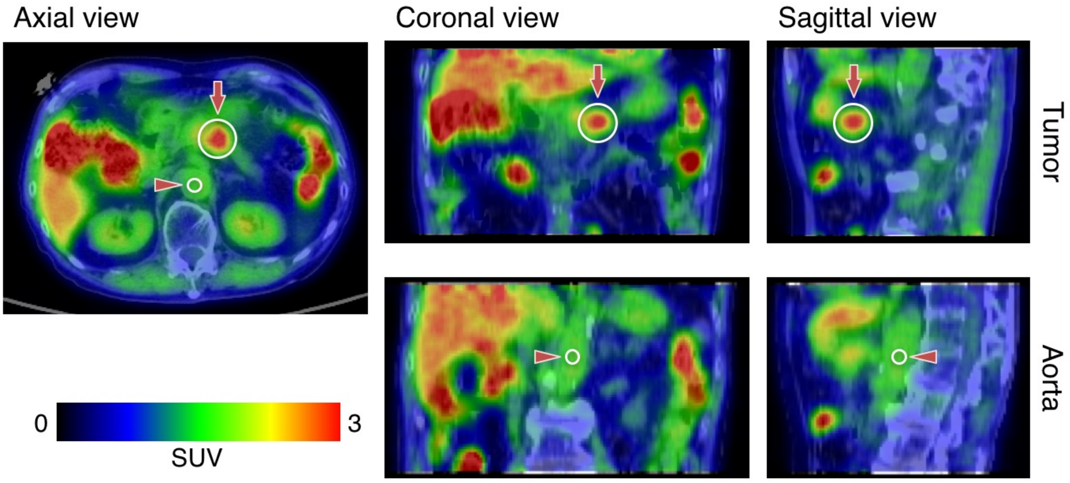

Fig. S1

Three-dimensional region of interest (ROI) replacement. SUVpeak of the tumor (arrows) was divided by SUVpeak of the aorta (arrowheads), and we defined tumor blood ratio using SUVpeak (TBRpeak) as the quantitative value. SUVpeak was defined as the mean value of the voxels within a fixed sphere of 1-cm^3 volume centered on the hottest area of the ROI over the tumor. SUV, standardized uptake value
