# Supplementary material for: [18F]FMISO PET/CT as a preoperative prognostic factor in patients with pancreatic cancer
Source: EJNMMI Res. 2019 May 9;9:39. doi: 10.1186/s13550-019-0507-8 (PMC6509312; doi:10.1186/s13550-019-0507-8)
Supplement: Supplementary file 2 — Table S1. Patients’ characteristics. (PDF 86 kb) [file 13550_2019_507_MOESM2_ESM.pdf]

**Table S1**

Patients' characteristics

| Patient | Sex | Age | Body Weight (kg) | Visi-bility | [18F]FMISO uptake | | Ope-ration? | Days between [18F]FMISO PET/CT and operation | Tissue acqured by | HIF1-α expression | | Recurrence after operation? | Recurrence days after operation | Arive? | Follow-up days |
|---|---|---|---|---|---|---|---|---|---|---|---|---|---|---|---|
| | | | | | TBR peak | Binary | | | | Category 0-3 | Binary | | | | |
| 1 | M | 77 | 53 | Yes | 1.77 | High | No | N/A | N/A | | | | | No | 329 |
| 2 | F | 75 | 39 | No | 1.08 | Low | Yes | 30 | Operation | 0 | Negative | Yes | 234 | No | 286 |
| 3 | M | 76 | 57 | No | 0.80 | Low | Yes | 13 | Operation | 1 | Negative | No | | Yes | 686 |
| 4 | M | 75 | 59 | No | 1.03 | Low | Yes | 6 | Operation | 1 | Negative | Yes | 332 | No | 746 |
| 5 | F | 76 | 45 | No | 1.11 | Low | Yes | 20 | Operation | 1 | Negative | Yes | 309 | No | 414 |
| 6 | M | 67 | 57 | No | 1.13 | Low | Yes | 5 | Operation | 3 | Positive | Yes | 353 | No | 587 |
| 7 | F | 60 | 49 | Yes | 2.19 | High | No | N/A | N/A | | | | | No | 39 |
| 8 | F | 67 | 58 | Yes | 1.48 | High | No | N/A | N/A | | | | | No | 346 |
| 9 | M | 67 | 66 | No | 1.04 | Low | Yes | 7 | Operation | 2 | Positive | Yes | 234 | Yes | 1101 |
| 10 | F | 73 | 48 | No | 0.89 | Low | Yes | 11 | Operation | 0 | Negative | Yes | 561 | Yes | 591 |
| 11 | M | 61 | 69 | Yes | 1.24 | Low | Yes | 14 | Operation | 3 | Positive | Yes | 441 | Yes | 869 |
| 12 | F | 69 | 47 | Yes | 1.70 | High | Yes | 25 | Operation | 3 | Positive | Yes | 60 | No | 415 |
| 13 | M | 74 | 61 | No | 1.09 | Low | Yes | 16 | Operation | 2 | Positive | No | | Yes | 869 |
| 14 | F | 60 | 58 | No | 1.02 | Low | Yes | 9 | Operation | 2 | Positive | No | | Yes | 850 |
| 15 | F | 81 | 52 | No | 1.15 | Low | Yes | 4 | Operation | 3 | Positive | Yes | 85 | No | 137 |
| 16 | M | 62 | 65 | Yes | 1.50 | High | Yes | 28 | Operation | 1 | Negative | Yes | 218 | No | 369 |
| 17 | M | 76 | 53 | No | 0.85 | Low | No | N/A | Biopsy | 1 | Negative | | | Yes | 477 |
| 18 | M | 78 | 57 | Yes | 1.51 | High | Yes | 2 | Operation | 2 | Positive | Yes | 246 | No | 437 |
| 19 | M | 73 | 40 | Yes | 1.27 | High | No | N/A | Biopsy | 2 | Positive | | | No | 209 |
| 20 | F | 69 | 53 | No | 1.22 | Low | Yes | 18 | Operation | 1 | Negative | No | | Yes | 358 |
| 21 | M | 77 | 60 | No | 1.06 | Low | Yes | 16 | Operation | 0 | Negative | No | | Yes | 310 |
| 22 | F | 74 | 49 | No | 1.17 | Low | Yes | 16 | Operation | 1 | Negative | No | | Yes | 283 |
| 23 | M | 58 | 80 | No | 1.20 | Low | Yes | 27 | Operation | 1 | Negative | No | | Yes | 296 |
| 24 | M | 62 | 74 | Yes | 1.19 | Low | Yes | 14 | Operation | 1 | Negative | Yes | 241 | Yes | 282 |
| 25 | F | 69 | 50 | No | 1.09 | Low | Yes | 25 | Operation | 3 | Positive | No | | Yes | 190 |

[18F]FMISO, [18F]fluoromisonidazole; TBRpeak, Tumor blood ratio using SUVpeak (SUVpeak of the tumor divided by SUVpeak of the aorta); SUV, Standardized uptake value; HIF, Hypoxia-inducible factor, N/A Not applicable.
