# Supplementary figures and images for: [18F]FMISO PET/CT as a preoperative prognostic factor in patients with pancreatic cancer
Source: EJNMMI Res. 2019 May 9;9:39. doi: 10.1186/s13550-019-0507-8 (PMC6509312; doi:10.1186/s13550-019-0507-8)

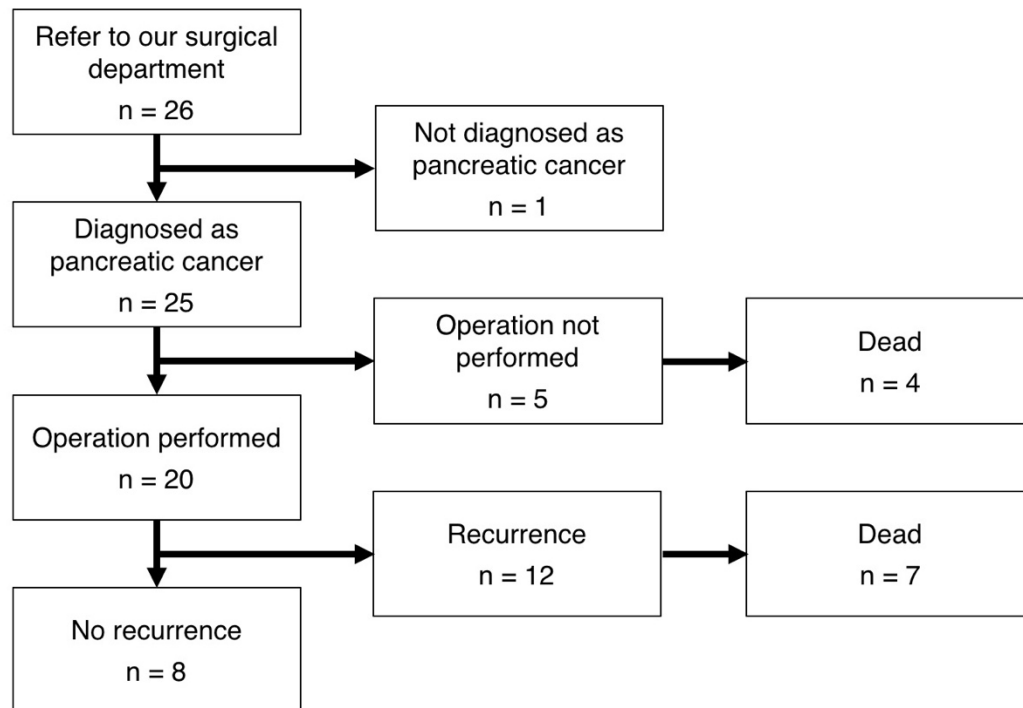

**Fig. S2**

Flow diagram of the patients

Supplement: Supplementary file 3 — Figure S2. Flow diagram of the patients. (PDF 155 kb) [file 13550_2019_507_MOESM3_ESM.pdf]
